# Enhanced Discussion on Reassessing Lipinski’s Rule of Five in the Era of AI-Driven Drug Discovery

**DOI:** 10.34172/apb.025.45948

**Published:** 2025-08-31

**Authors:** Uppula Purushotham

**Affiliations:** Department of Chemistry, Guru Nanak Institutions Technical Campus, School of engineering and Technology Hyderabad, Telangana, India

## To Editor,

 Artificial intelligence (AI) is redefining drug discovery, prompting reevaluation of established frameworks like Lipinski’s Rule of Five (Ro5). Although Ro5 has been a key component in the prediction of oral bioavailability, its strict use in the AI era continues the danger of inhibiting innovation by omitting promising compounds that challenge established norms.

 Young recently examined Ro5’s limitations and historical context in a 2023 Expert Opinion on Drug Discovery article.^1^ The paper underscores that the rule’s 90th-centile thresholds are often misinterpreted as absolute limits. This misinterpretation has led to the exclusion of viable compounds, especially in emerging therapeutic areas that require larger molecules such as protein-protein interaction inhibitors, PROTACs, and macrocycles. Young highlights that molecular weight (MW), the most frequently violated Ro5 parameter, is increasingly irrelevant in modern drug design, as evidenced by the success of beyond Ro5 (bRo5) drugs with MW > 700 Da. These molecules leverage advanced design principles, such as conformational flexibility (chameleonicity) and transporter-mediated uptake, to achieve bioavailability.^1^

**Figure 1:**
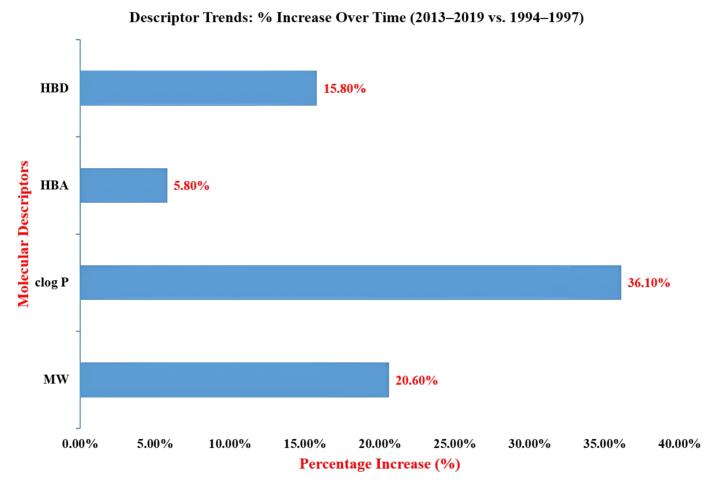
Percentage increase in key molecular descriptors of FDA-approved oral drugs between 1994-1997 and 2013-2019

Figure 1 illustrates the relative increase in molecular complexity over two decades. Notably, lipophilicity (clogP) exhibited the highest percentage increase (~36%), reflecting a growing tolerance for higher lipophilicity in modern drugs. Additionally, MW increased by more than 20%, suggesting a tendency toward bigger and more complex molecules. Higher molecular weight and lipophilicity have been clearly accepted; from 2013 to 2019, around 40% of new medications violated at least one Ro5 rule.^2^

 Selected oral medications that surpass conventional Ro5 parameters, particularly in MW and lipophilicity (clogP), are shown in Table 1. Despite exhibiting several violations, several of these compounds including Venetoclax, Pibrentasvir, and Ledipasvir have acquired oral bioavailability using sophisticated formulation techniques. These include stabilizing methods based on polymers, hot-melt extrusion and amorphous solid dispersions, which improve absorption and solubility. The incorporation of the prodrug fostamatinib, which is activated in vivo, is an alternate method of chemical modification to get beyond permeability restrictions. Collectively, these examples demonstrate how formulation is more important than Ro5 in the creation of bRo5 medication candidates.^2^

**Table 1:** Representative bRo5 Oral drugs with Ro5 violations and corresponding formulation strategies

**Drug**	**MW (Da)**	**clogP**	**Exceeding descriptors**	**Formulation strategy**
Venetoclax	868.5	6.76	MW, clogP	Copovidone based amorphous dispersion used
Pibrentasvir	1113	5.95	MW, clogP,	Amorphous dispersion with poly ethylene glycol
Ledipasvir	889	5.98	MW, clogP	Amorphous spray-dried dispersion
Fostamatinib	580.5	2.78	MW, HBA = 13	Prodrug converted in vivo
Ombitasvir	894.1	5.72	MW, clogP	Hot-melt extrusion with inhibitors and stabilizers

 AI-driven platforms are uniquely positioned to exploit these differences. By integrating high-throughput physicochemical measurements (e.g., chromatographic log D, EPSA for chameleonicity) and predictive models. Artificial intelligence can improve drugs for effective lipophilicity and permeability parameters that are more predictive of success than Ro5’s static thresholds.^3^ For instance, the AbbVie Multi-Parameter Scoring (MPS) function, which prioritizes log D 7.4 ≈ 3, low rotatable bonds, and minimal aromatic rings, has demonstrated efficacy in guiding bRo5 drug development. Similarly, AI can harness natural product-inspired design, as noted by Young,^1^ to exploit evolutionary-refined transporter interactions, bypassing Ro5 constraints altogether.

 With the rise of complex molecules, AI-driven models have evolved to handle the unique challenges posed by bRo5 scaffolds. That includes the compounds conformational flexibility, non-linear structure activity relationships and formulation-dependent pharmacokinetics.^4^ Recent developments in graph neural networks (GNNs), Transformer-based models, and attention mechanisms have shown superior performance in learning molecular representations for bRo5 libraries. Several studies provide validation benchmarks for such models here discussed few models.

 Using 2D descriptors, Poongavanam et al^5^ created random forest models to predict the passive permeability of macrocycles, with an accuracy of about 85% and an MCC of about 0.60. Only for stiff compounds with a Kier flexibility index ≤ 10 did 3D models enhance prediction. In order to predict cyclic peptide permeability, a study also presented a multimodal deep learning architecture that combined a transformer and a graph convolutional network. This resulted in an accuracy of roughly 0.82 and an AUC of 0.87, which is a notable advancement for modeling bRo5-like macrocyclic compounds.^6^

 Representative bRo5 pharmacological modalities are categorized in Table 2 according to their structural class and common Ro5 breaches. Despite frequently exceeding several parameters, such as MW, HBD, HBA, and topological polar surface area (TPSA). The macrocyclic medications, like pibrentasvir and cyclosporine A, have the advantage of conformational rigidity, which promotes membrane permeability. Because of their high molecular weight and structural flexibility, PROTACs (like ARV-110) have poor passive permeability. Because they resemble peptides, peptidomimetics, such as semaglutide, usually exceed MW and HBD limits. The amphiphilic hybrids like Venetoclax have high clogP and MW, requiring sophisticated formulation techniques. The diversity of bRo5 space and the requirement for customized design and delivery techniques are highlighted by this classification.^2^

**Table 2:** Representative categories of bRo5 molecules with common rule-of-five violations

**Molecule Type**	**Examples**	**Ro5 Violations**
Macrocycles	Cyclosporine A, Pibrentasvir	MW, HBA, HBD, TPSA
PROTACs	ARV-110, ARV-471	MW > 1000, high rotatability
Peptidomimetics	Semaglutide, Bortezomib	MW, HBD, PSA
Amphiphilic Hybrids	Venetoclax, Entrectinib	clogP, MW

 The general ability of conventional AI models trained primarily on Ro5-compliant datasets is challenged by changes in important molecular descriptors among bRo5 substances, as shown in Table 3. Chemically diverse, bRo5-specific training sets and sophisticated 3D-aware architectures like transformers or graph neural networks are required because increases in molecular weight and flexibility frequently lead to extrapolation errors and decreased QSAR performance. Furthermore, static descriptors (like LogP) and traditional ADMET models are unable to convey the complexity introduced by phenomena like chameleonic behavior and formulation reliance. When AI models are utilized in place of physicochemical criteria, they run the risk of overfitting to narrow chemical profiles and producing erroneous predictions, especially for underrepresented scaffolds like macrocycles or polar entities.

**Table 3:** Descriptor shifts in bRo5 space and implications for AI model adaptation

**Descriptor shift**	**Effect on traditional models**	**Required AI adaptation**
Molecular weight	Poor extrapolation from Ro5-trained datasets	Need bRo5-specific training sets
Flexibility (rot. bonds)	Decreased QSAR predictivity	Use of 3D-aware models (e.g., GNNs, transformers)
Chameleonic behavior	Misleading LogP/LogD assumptions	Include dynamic conformer libraries or solvent models
Formulation dependency	ADMET prediction failure	Integrate formulation metadata as co-variables

 The Ro5 should be reconsidered as an adaptable, AI-informed framework for modern drug development. Rather than blindly excluding compounds that violate Ro5, the studies should prioritize real-world data like experimental permeability, transporter effects, and in vivo performance over simplistic rules. AI can help by identifying promising outliers (e.g., natural product-inspired molecules) and optimizing compounds using advanced descriptors like chameleonicity and 3D polarity.^7^ The field needs a smarter definition of drug-likeness, one that balances predictive modeling with biological reality. Moving forward, the investigator advocates for collaborative efforts to refine these guidelines, ensuring they enable rather than restrict innovation in drug design.

## Conclusion

 In the evolving landscape of drug discovery, rigid adherence to the Ro5 risks overlooks promising therapeutic candidates, particularly those revealed through AI-driven innovation. Drug-likeness is being redefined by data-rich methods and new descriptors like dynamic polarity and chameleonicity. The researchers need to switch to evidence-based, flexible frameworks instead of rigid cutoffs. By modernizing Ro5 through the lens of AI and experimental validation, the researchers can retain its foundational value while unlocking broader chemical space for next-generation therapeutics.

## Competing Interests

 None declared.

## Ethical Approval

 Not applicable.
